# Biomarker Testing, Imaging, and Outcomes in Cardiology-Referred Acute Breathlessness: A Retrospective Clinical Audit

**DOI:** 10.7759/cureus.119147

**Published:** 2026-11-05

**Authors:** Jishel Mathews, Rana Kashif Riaz, Ayesha Kazi, Shamim Rahman

**Affiliations:** 1 Internal Medicine, Sandwell and West Birmingham NHS Trust, Birmingham, GBR; 2 Cardiology, Sandwell and West Birmingham NHS Trust, Birmingham, GBR

**Keywords:** acute breathlessness, acute coronary syndrome (acs), ct pulmonary angiogram (ctpa), d-dimer, dyspnea, high-sensitivity troponin i, left ventricular systolic dysfunction, nt-probnp, pulmonary embolism (pe), transthoracic echocardiogram

## Abstract

Background

Breathlessness is a leading cause of emergency hospital attendances, and its multifactorial nature often reflects overlapping cardiopulmonary pathologies such as pulmonary embolism (PE), heart failure (HF), and acute coronary syndrome (ACS). Computed tomography pulmonary angiography (CTPA) is frequently used, often without documented pretest probability assessments. This audit primarily assessed the diagnostic yield of CTPA for suspected PE against the Royal College of Radiologists (RCR) standard of ≥15% and secondarily described biomarker testing, imaging, and outcomes across the PE, HF, and ACS pathways in patients referred to cardiology with acute breathlessness.

Methods

We conducted a retrospective audit of 70 adults referred to cardiology with breathlessness through the electronic patient record or multiprofessional team list (MPTL) referral systems at a district general hospital in the United Kingdom between March 1 and June 30, 2025. Biomarker testing for D-dimer, N-terminal pro-B-type natriuretic peptide (NT-proBNP), and high-sensitivity troponin I (hs-TnI), together with imaging and outcomes, was reviewed and described across the PE, HF, and ACS pathways, with diagnostic yields restricted to CTPA for suspected PE and coronary angiography for suspected ACS. Yields were reported with Wilson 95% CIs and compared using exploratory Fisher’s exact tests. Additional analyses examined biomarker concentrations by outcome and overlap in biomarker and imaging use.

Results

Seventy patients were included (median age, 69.5 years; 52.9% male). D-dimer, NT-proBNP, and hs-TnI were tested in 68.6%, 58.6%, and 81.4% of patients, respectively. Overall, seven of 54 CTPAs performed for suspected PE were positive (13.0%, 95% CI, 6.4-24.4), numerically below the RCR benchmark of ≥15%. A clinically relevant alternative diagnosis was reported in 44.4% (target, ≥50%), and pretest probability was documented in 22.2% (target, 100%). PE yield was 15.0% (6/40) among patients with an elevated D-dimer, 0% (0/4) among those with a normal D-dimer, and 10.0% (1/10) among those without D-dimer testing (p = 1.000 for both comparisons). Left ventricular systolic dysfunction (LVSD) was found on transthoracic echocardiography in 40.0% (8/20) of patients with elevated NT-proBNP, 23.1% (3/13) of those with non-elevated NT-proBNP, and 26.3% (5/19) of those without testing (p = 0.456 and p = 0.501, respectively). Of 47 patients with elevated hs-TnI, 10 (21.3%) underwent coronary angiography for suspected ACS, with percutaneous coronary intervention performed in four (40.0%). Overall, 29 of 57 patients tested (50.9%) had hs-TnI levels above the sex-specific 99th-percentile upper reference limit.

Conclusions

Cardiology-referred breathlessness frequently involved multiple biomarker and imaging pathways during the same admission. CTPA yield, alternative diagnosis rates, and pretest probability documentation fell below RCR standards. LVSD was more frequent with elevated NT-proBNP, although differences were not statistically significant. No hs-TnI comparison was possible because all patients undergoing angiography for suspected ACS had elevated levels. Given the small, single-center sample and selective imaging, these findings do not establish diagnostic accuracy or pathway effectiveness. Larger multicenter studies are needed. The findings support reviewing pretest probability documentation and investigation decisions, followed by reaudit against RCR standards.

## Introduction

Dyspnea, or breathlessness, is one of the most common presenting symptoms in acute medicine [1,2], accounting for over 1.3 million (4.9%) unplanned ED attendances in England in 2025-2026 and ranking as the third most common chief complaint after abdominal pain and chest pain [1]. The complex and undifferentiated nature of its etiology may reflect multiple overlapping cardiopulmonary causes, such as pulmonary embolism (PE), heart failure (HF), and acute coronary syndrome (ACS), often prompting a cardiology referral.

National and international guidelines recommend a structured approach to investigation: pretest probability assessment using the Wells score, with the pulmonary embolism rule-out criteria (PERC) and D-dimer testing reserved for lower-probability patients before computed tomography pulmonary angiography (CTPA) for suspected PE [3-8]; natriuretic peptide testing to guide echocardiography for suspected HF [9-11]; and high-sensitivity troponin testing to risk-stratify patients with suspected ACS [12]. Despite these recommendations, CTPA is frequently requested without a documented pretest probability assessment [13-15]. The Royal College of Radiologists (RCR) has proposed a benchmark diagnostic yield of at least 15% for CTPA performed for suspected PE, alongside standards for pretest probability documentation and reporting of alternative diagnoses [16].

To our knowledge, few UK studies have concurrently quantified diagnostic yield and outcomes across the PE, HF, and ACS pathways in a single cardiology-referred cohort; pathway analyses are more frequently organized around individual diagnoses than presenting symptoms [17].

The primary objective of this clinical audit was to determine the diagnostic yield of CTPA for suspected PE against the RCR standard of ≥15% in cardiology-referred patients with acute breathlessness. Secondary objectives were to describe biomarker testing, imaging, and outcomes in the PE, HF, and ACS pathways and to measure compliance with the remaining RCR audit standards for pretest probability documentation and reporting of a clinically relevant alternative diagnosis. Exploratory analyses compared diagnostic yield by biomarker status and described the primary attributed causes of breathlessness, overlap in biomarker testing and imaging, and biomarker concentrations by outcome.

The preliminary findings from this audit were previously presented as a conference abstract at the British Cardiovascular Society Annual Conference on June 1-3, 2026, and published in abstract form in the Heart conference supplement [18].

## Materials and methods

Audit setting, registration, and standards

This retrospective clinical audit took place at Midland Metropolitan University Hospital, Sandwell and West Birmingham NHS Trust, a district general hospital in the United Kingdom. The audit was registered with the Trust’s Clinical Audit Department (registration number 3091.1).

Practice was audited against the RCR CTPA audit standards [16]: diagnostic yield of CTPA for suspected PE (target, ≥15%); reporting of a clinically relevant alternative diagnosis on CTPA (target, ≥50%); and pretest probability assessment before CTPA, measured by documentation in the electronic patient record (EPR; target, 100%). National Institute for Health and Care Excellence and European Society of Cardiology (ESC) guidance for PE, HF, and ACS [6,7,9,10,12], which sets no numerical targets for the investigations audited here, was used as a reference for describing investigation practices rather than as an audit standard.

Audit population

Adults aged 18 years or older who were admitted between March 1 and June 30, 2025, were eligible for inclusion if breathlessness was a primary or secondary presenting symptom and they were referred to cardiology through the EPR or multiprofessional team list (MPTL) referral systems. Breathlessness was identified when documented during admission up to the time of referral, including in ED triage or assessment, medical clerking, ward reviews, or the cardiology referral itself. Patients were excluded if breathlessness was not a presenting symptom, if they were younger than 18 years, if they were admitted outside the audit period, or if their medical records were incomplete. All 440 cardiology referrals made through the EPR and MPTL during the audit period were retrieved and screened. Of these, 366 were excluded, and 74 were eligible. After four duplicate records were excluded, 70 patients were included.

Data collection 

Data were extracted from the EPR by three authors using a standardized extraction template, and all records were re-reviewed in full by the lead authors. Extracted variables included patient demographics, comorbidities, biomarker results (D-dimer, N-terminal pro-B-type natriuretic peptide (NT-proBNP), and high-sensitivity troponin I (hs-TnI)), imaging investigations (CTPA, transthoracic echocardiography (TTE), and coronary angiography) and their indications, outcomes (PE on CTPA, left ventricular systolic dysfunction (LVSD) on TTE, and percutaneous coronary intervention (PCI)), and the primary attributed cause of breathlessness.

Biomarker results were linked to imaging when both occurred during the same admission, and the timing of each biomarker measurement relative to the corresponding imaging was recorded. When repeated measurements were available, the peak value before the corresponding imaging was used, or the peak value during the admission if no imaging was performed. The primary attributed cause was assigned by the lead author based on the combined clinical record, comprising consultant-led ward reviews, specialty reviews, and discharge summaries. Biomarkers not measured during the admission were categorized as not tested, without imputation.

Biomarker thresholds and outcome definitions

Biomarkers were categorized as elevated, normal (or non-elevated for NT-proBNP), or not tested. D-dimer was measured using the HemosIL D-Dimer HS 500 assay (Werfen, Bedford, MA, USA) and reported in ng/mL fibrinogen-equivalent units (FEU). The laboratory routinely applies an age-adjusted cutoff (≥500 ng/mL, or ≥age × 10 ng/mL for patients aged ≥50 years) [5], which was used to classify results. NT-proBNP was measured using the Alere NT-proBNP assay for Alinity i (Abbott Laboratories, Abbott Park, IL, USA). Elevation was defined retrospectively using the 2026 ESC age-related acute thresholds (>450, >900, and >1,800 pg/mL for ages <50, 50-74, and >74 years, respectively) [19]. hs-TnI was measured using the STAT High Sensitive Troponin-I assay for Alinity i (Abbott Laboratories). Results ≥5 ng/L were classified as elevated because they were above the laboratory reference range. The manufacturer’s sex-specific 99th-percentile upper reference limits (URLs; 15.6 ng/L for females and 34.2 ng/L for males) are reported for comparison.

Outcomes were defined as PE reported on CTPA performed for suspected PE; LVSD, defined as left ventricular ejection fraction (LVEF) <50% on TTE, consistent with the 2026 ESC classification [19]; and PCI following coronary angiography performed for suspected ACS, used as a proxy treatment outcome; all angiography and PCI procedures were performed on site. LVEF was taken from the formal echocardiography report (visual estimate or Simpson’s biplane method), and LVSD included both new and previously known cases. One CTPA performed for suspected chronic thromboembolic pulmonary hypertension and five angiograms performed for other indications were excluded from the pathway yields. Additionally, PE detected on imaging other than CTPA was excluded from the CTPA yield. A clinically relevant alternative diagnosis on CTPA excluded isolated mild atelectasis and incidental findings.

Statistical analysis and visualization 

Descriptive results are reported as counts and percentages of the relevant denominator for each pathway. Outcomes were expressed as proportions with 95% CIs calculated using the Wilson score method. Exploratory comparisons, which were not prespecified, compared the yield in the elevated group separately with that in the normal (or non-elevated) and not-tested groups using two-sided Fisher’s exact tests, with p < 0.05 considered statistically significant. Distributions of raw biomarker values by outcome were summarized as medians with IQRs and presented as simple box-and-whisker plots on a logarithmic scale. Overlap in biomarker testing and imaging was visualized using three-set Venn diagrams constructed in R, version 4.6.1 (R Foundation for Statistical Computing, Vienna, Austria), with the VennDiagram package (version 1.8.2). All statistical analyses were carried out using IBM SPSS Statistics for Windows, version 27.0 (released 2019; IBM Corp., Armonk, NY, USA).

For patients with a biomarker result but no CTPA, 90-day readmissions, venous thromboembolism (VTE) diagnoses, and new anticoagulation were identified from their EPRs through clinical reviews and discharge summaries.

## Results

Seventy patients were included in the audit (median age, 69.5 years; range, 19-93 years). Demographics and comorbidities are summarized in Table 1. Anemia was present in 31 patients (44.3%), a recognized contributor to breathlessness.

**Table 1:** Baseline demographics and comorbidities (n = 70). ^a^ Anemia was defined as hemoglobin <130 g/L in males and <120 g/L in females, per World Health Organization criteria [20]. eGFR: estimated glomerular filtration rate; Hb: hemoglobin; HF: heart failure; LVSD; left ventricular systolic dysfunction

Characteristic	Value	% of cohort
Median age, years (IQR; range)	69.5 (56.5-79.3; 19-93)	-
Sex, male	37	52.9
Sex, female	33	47.1
Ischemic heart disease	27	38.6
Known HF/LVSD before admission	17	24.3
Atrial fibrillation	9	12.9
Chronic lung disease	19	27.1
Active malignancy	6	8.6
eGFR <60 mL/min/1.73m²	24	34.3
Anemia (Hb below sex-specific threshold)ᵃ	31	44.3

The primary attributed cause of breathlessness is shown in Table 2. Respiratory or other infection was the most commonly identifiable etiology (14/70, 20.0%), followed closely by HF/LVSD (13/70, 18.6%). No clear cause was identified in five patients (7.1%). A significant minority (19/70, 27.1%) had multiple documented contributing diagnoses; hence, Table 2 reflects the single most clinically dominant cause per patient. Four patients (5.7%) died during the index admission, two from PE and two from HF, as recorded on the Medical Certificate of Cause of Death, and at least two additional patients died within 90 days of the index admission.

**Table 2:** Primary attributed cause of dyspnea (n = 70). ^a^ One PE was diagnosed on CT angiography rather than CTPA. ACS: acute coronary syndrome; CTPA: computed tomography pulmonary angiography; HF: heart failure; LVSD: left ventricular systolic dysfunction; PE: pulmonary embolism

Primary attributed cause of dyspnea	n	%
Respiratory or other infection (including infective exacerbation of airway diseases)	14	20
HF/LVSD	13	18.6
PE^a^	8	11.4
Other	8	11.4
Ischemic heart disease/ACS	6	8.6
No cause identified	5	7.1
Arrhythmia/conduction disorder	5	7.1
Pulmonary hypertension/cor pulmonale	5	7.1
Valvular heart disease (including infective endocarditis)	4	5.7
Noninfective airway disease	2	2.9

Biomarker testing and subsequent imaging according to biomarker status are summarized in Table 3. hs-TnI was measured in 81.4% (57/70) of patients, D-dimer in 68.6% (48/70), and NT-proBNP in 58.6% (41/70), with elevated results in 82.5% (47/57), 87.5% (42/48), and 56.1% (23/41), respectively.

**Table 3:** Investigation utilization of biomarkers (n = 70). Imaging performed refers to the corresponding investigation for each biomarker: CTPA for D-dimer, TTE for NT-proBNP, and coronary angiography for hs-TnI. This includes investigations performed for reasons other than suspected PE, HF, or ACS. ACS: acute coronary syndrome; CTPA: computed tomography pulmonary angiography; HF: heart failure; hs-TnI: high-sensitivity troponin I; NT-proBNP: N-terminal pro-B-type natriuretic peptide; PE: pulmonary embolism; TTE: transthoracic echocardiography

Biomarker	Elevated, n (imaging performed, n/N, %)	Normal/non-elevated, n (imaging performed, n/N, %)	Not tested, n (imaging performed, n/N, %)
D-dimer	42 (40/42, 95.2%)	6 (4/6, 66.7%)	22 (11/22, 50.0%)
NT-proBNP	23 (20/23, 87.0%)	18 (13/18, 72.2%)	29 (19/29, 65.5%)
hs-Troponin I	47 (15/47, 31.9%)	10 (0/10, 0%)	13 (0/13, 0%)

The overlap in biomarker testing and imaging is shown in Figures 1, 2. Figure 1 highlights that hs-TnI was the most frequently measured biomarker and was tested in 50 of the 55 patients (90.9%) who had two or more biomarkers measured. D-dimer was measured in isolation only once, with 47 of 48 (97.9%) D-dimer tests performed alongside at least one other biomarker. This pattern is consistent with biomarkers being used together in the assessment of undifferentiated breathlessness, which may reflect the etiological ambiguity it poses.

**Figure 1:**
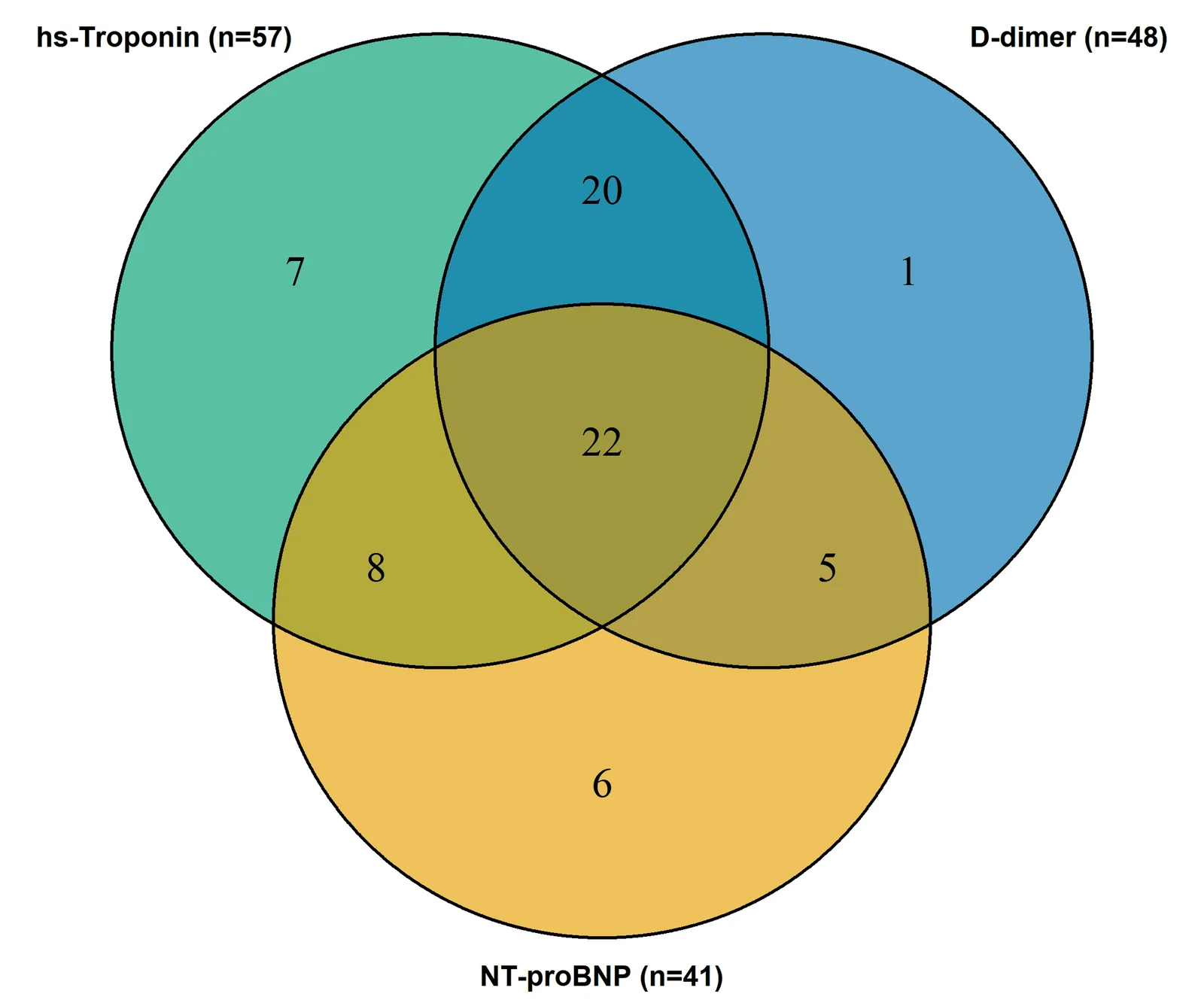
Overlap of biomarker testing. Values show the number of patients tested for each biomarker or combination of biomarkers. One patient had none of the three biomarkers tested (n = 70 total). hs-Troponin: high-sensitivity troponin I; NT-proBNP: N-terminal pro-B-type natriuretic peptide. Image created in R, version 4.6.1 (R Foundation for Statistical Computing, Vienna, Austria) using the VennDiagram package (version 1.8.2).

**Figure 2:**
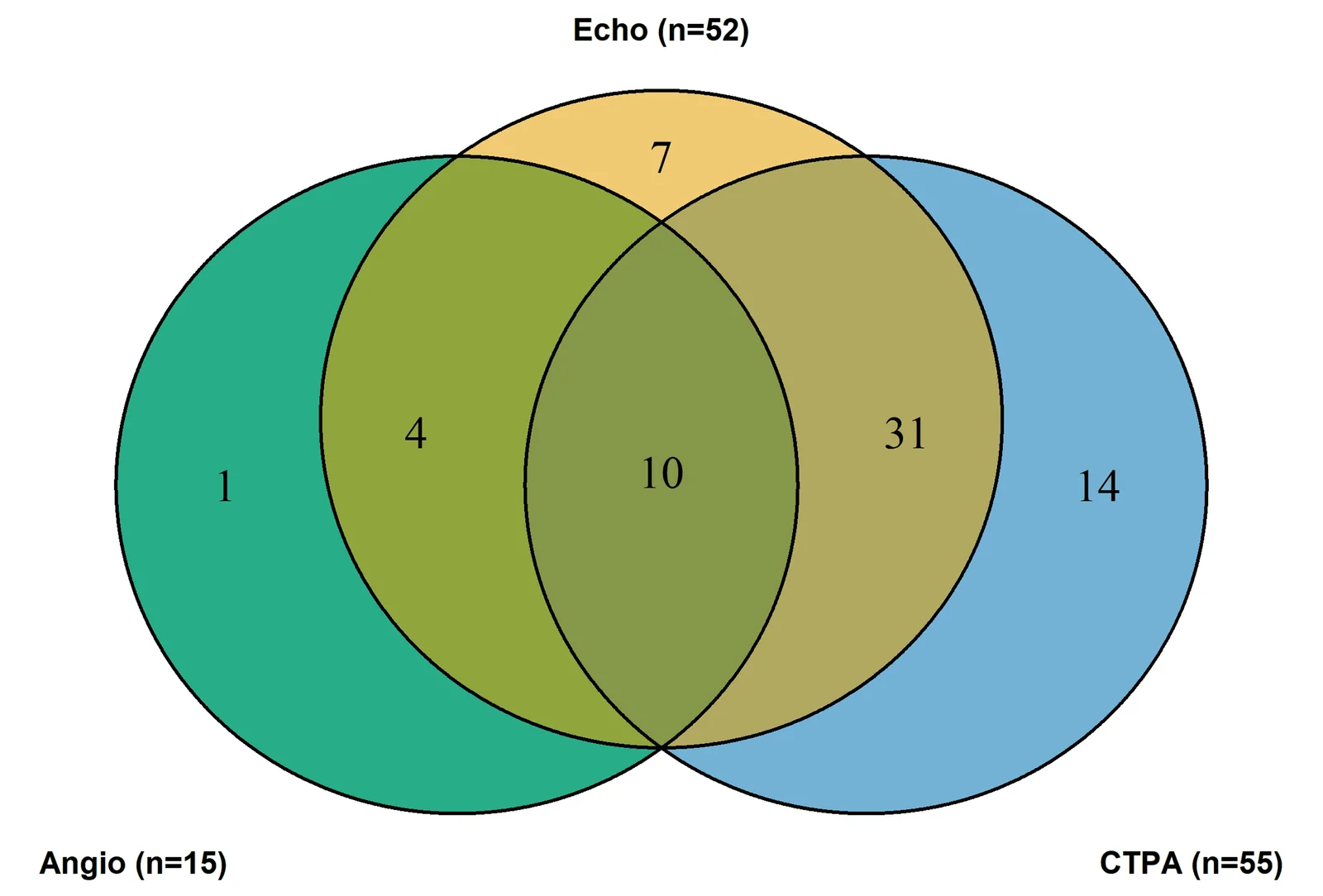
Overlap of imaging investigations. Values show the number of patients who underwent each imaging investigation or combination of investigations, including investigations performed for reasons other than suspected PE, HF, or ACS. Three patients had none of the three imaging investigations performed (n = 70 total). ACS: acute coronary syndrome; angio: coronary angiography; CTPA: computed tomography pulmonary angiography; HF: heart failure; echo: transthoracic echocardiography; PE: pulmonary embolism Image created in R, version 4.6.1 (R Foundation for Statistical Computing, Vienna, Austria) using the VennDiagram package (version 1.8.2).

Figure 2 demonstrates that TTE and CTPA were the most frequently co-performed imaging modalities (41/70, 58.6%), indicating that the PE and HF pathways were often investigated during the same admission. Additionally, coronary angiography was performed in isolation only once, with the majority (10/15, 66.7%) performed alongside both CTPA and TTE. Notably, no patient underwent both CTPA and angiography without TTE. As the order of imaging investigations was not extracted, these patterns reflect co-occurrence within the same admission rather than a specific sequence.

Outcomes, defined as PE on CTPA, LVSD on TTE, and PCI following angiography, are summarized in Table 4. Overall, of 54 CTPAs performed for suspected PE, seven confirmed PE (13.0%, 95% CI 6.4-24.4), numerically below the RCR benchmark of ≥15%. A clinically relevant alternative diagnosis was reported in 24 (44.4%; target ≥50%), and a pretest probability assessment was documented before CTPA in 12 (22.2%; target 100%), of whom nine were classified as PE likely and three as PE unlikely. On subgroup analysis, PE yield was 15.0% (6/40, 95% CI 7.1-29.1) among patients with an elevated D-dimer, 0% (0/4, 95% CI 0-49.0) among those with a normal D-dimer, and 10.0% (1/10, 95% CI 1.8-40.4) among those without D-dimer testing. Yield was numerically highest among patients with an elevated D-dimer, matching the RCR benchmark, although comparisons between groups were not statistically significant (p = 1.000 for both). Of the 14 patients undergoing CTPA with a normal or untested D-dimer, a Wells score was documented in five, all of whom were classified as PE likely, consistent with proceeding directly to CTPA. One further PE was identified incidentally on a CT aortic angiogram and was not included in these analyses.

**Table 4:** Diagnostic yield from biomarker testing across the PE, HF, and ACS pathways. The outcome was defined as PE on CTPA, LVSD on TTE, and PCI following coronary angiography. ᵃ CTPAs performed for suspected PE (54 of 55; one performed for suspected chronic thromboembolic pulmonary hypertension was excluded). ᵇ Angiography performed for suspected ACS (10 of 15; five performed for valve workup, left ventricular dysfunction workup, or syncope were excluded); no patient undergoing angiography for suspected ACS had a normal or untested hs-TnI. ᶜ Of the 10 patients, eight had hs-TnI above the sex-specific 99th-percentile URL, including all four who underwent PCI. ACS: acute coronary syndrome; CTPA: computed tomography pulmonary angiography; HF: heart failure; hs-TnI: high-sensitivity troponin I; LVSD: left ventricular systolic dysfunction; NT-proBNP: N-terminal pro-B-type natriuretic peptide; PCI: percutaneous coronary intervention; PE: pulmonary embolism; TTE: transthoracic echocardiography; URL: upper reference limit

Pathway	Biomarker	Imaging	Overall outcome, n/N (%)	Elevated outcome, n/N (%)	Normal/non-elevated outcome, n/N (%)	Not-tested outcome, n/N (%)
PE	D-dimer	CTPA^a^	7/54 (13.0%)	6/40 (15.0%)	0/4 (0%)	1/10 (10.0%)
HF	NT-proBNP	TTE	16/52 (30.8%)	8/20 (40.0%)	3/13 (23.1%)	5/19 (26.3%)
ACS	hs-TnI	Coronary angiography^b^	4/10 (40.0%)	4/10 (40.0%)^c^	0 - N/A	0 - N/A

Among patients undergoing TTE, LVSD was identified in 16/52 (30.8%, 95% CI 19.9-44.3), of whom 14 had new diagnoses and two had previously known diagnoses. Elevated NT-proBNP was accompanied by a higher rate of LVSD detected on TTE (40.0%, 8/20, 95% CI 21.9-61.3) than non-elevated NT-proBNP (23.1%, 3/13, 95% CI 8.2-50.3) or absent NT-proBNP testing (26.3%, 5/19, 95% CI 11.8-48.8); neither comparison was statistically significant (p = 0.456 and p = 0.501, respectively).

Of 47 patients with elevated hs-TnI, 10 (21.3%) underwent coronary angiography for suspected ACS, of whom four (40.0%, 95% CI 16.8-68.7) underwent PCI. As all patients undergoing angiography for suspected ACS had elevated hs-TnI, no comparison by troponin status was possible. Additionally, 29 of 57 patients tested (50.9%) had hs-TnI above the sex-specific 99th-percentile URL, including all four who underwent PCI. Of the six patients with ischemic heart disease/ACS as the primary attributed cause, four underwent PCI, and two were managed medically. It is important to consider that NT-proBNP and hs-TnI were evaluated against only TTE-detected LVSD and PCI, respectively. Hence, these figures likely underestimate the true positive rates for HF and ACS more broadly, as conditions such as HF with preserved ejection fraction, valvular disease, or medically managed myocardial infarction, such as plaque erosion, spontaneous coronary artery dissection, or myocardial infarction with nonobstructive coronary arteries (MINOCA), would not be captured by these specific measures.

Table 5 summarizes yields with 95% CIs by biomarker status, together with exploratory comparisons using Fisher’s exact test. Comparator groups were small (four patients with a normal D-dimer and 13 with non-elevated NT-proBNP), and no comparison was possible for hs-TnI because all patients undergoing angiography for suspected ACS had an elevated result. Using the sex-specific 99th-percentile URL instead, only two of these 10 patients were below the limit, neither of whom underwent PCI.

**Table 5:** Diagnostic outcomes with 95% CI and exploratory comparison. The yields are expressed as proportions with 95% CIs calculated using the Wilson score interval and stratified by biomarker status. PE yield is shown for CTPAs performed for suspected PE (n = 54), and LVSD yield is shown for all TTEs (n = 52). CTPA: computed tomography pulmonary angiography; LVSD: left ventricular systolic dysfunction; NT-proBNP: N-terminal pro-B-type natriuretic peptide; PE: pulmonary embolism; TTE: transthoracic echocardiography

Comparison	Overall yield (95% CI)	Yield, normal/non-elevated (95% CI)	Yield, not tested (95% CI)	Yield, elevated (95% CI)	Fisher’s p, elevated vs normal/non-elevated	Fisher’s p, elevated vs not tested
PE (CTPA) by D-dimer status	13.0% (6.4-24.4)	0% (0-49.0)	10.0% (1.8-40.4)	15.0% (7.1-29.1)	1.000	1.000
LVSD (TTE) by NT-proBNP status	30.8% (19.9-44.3)	23.1% (8.2-50.3)	26.3% (11.8-48.8)	40.0% (21.9-61.3)	0.456	0.501

These yields and their 95% CIs are further illustrated in Figure 3, alongside the RCR benchmark pertaining to the PE pathway.

**Figure 3:**
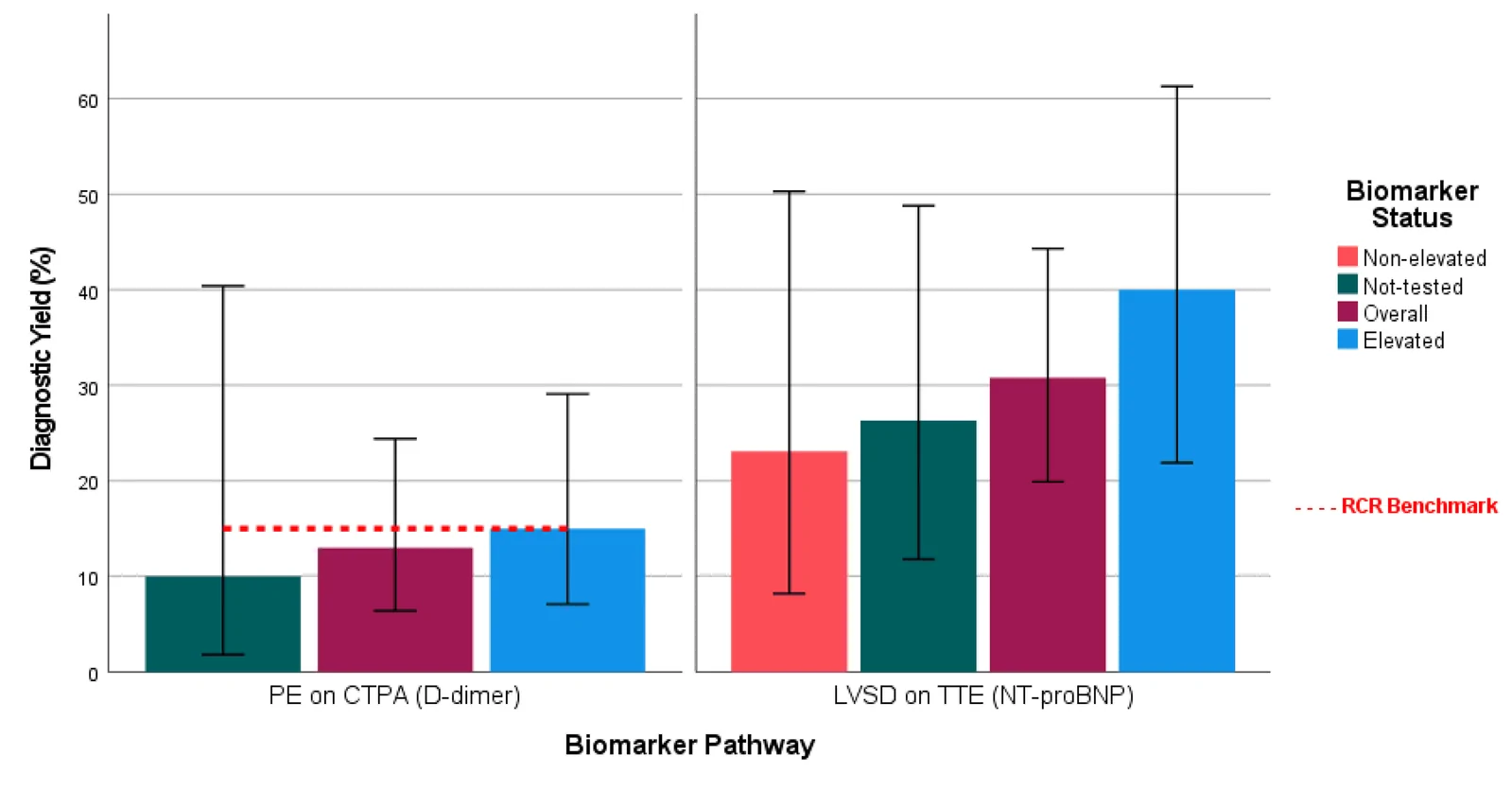
Diagnostic yield by biomarker status. Bars show diagnostic yield by biomarker status, with 95% Wilson CIs expressed as error bars. PE yield is shown for CTPAs performed for suspected PE (n = 54), and LVSD yield is shown for all TTEs (n = 52). The dashed line marks the RCR benchmark of 15% CTPA yield for suspected PE [16]. The normal D-dimer group is not shown because no patient had PE (0/4). CTPA: computed tomography pulmonary angiography; LVSD: left ventricular systolic dysfunction; NT-proBNP: N-terminal pro-B-type natriuretic peptide; PE: pulmonary embolism; RCR: Royal College of Radiologists; TTE: transthoracic echocardiography Image created in IBM SPSS Statistics for Windows, version 27.0 (released 2019; IBM Corp., Armonk, NY, USA).

Biomarker concentrations by outcome within each pathway are shown in Figure 4A-4C. Among patients undergoing CTPA for suspected PE, the median D-dimer level was 16,298.5 ng/mL FEU (IQR 3,140.0-57,764.0) in those with PE and 1,269.5 ng/mL FEU (IQR 990.0-2,018.0) in those without PE. An analogous pattern was observed for NT-proBNP, with median levels of 7,713 pg/mL (IQR 551-25,092) in patients with LVSD and 1,405 pg/mL (IQR 171-3,975) in those without LVSD. Similarly, among patients undergoing angiography for suspected ACS, the median hs-TnI level was 1,997.4 ng/L (IQR 130.6-4,144.1) in those who underwent PCI and 28.4 ng/L (IQR 8.8-86.3) in those who did not. Nonetheless, the ranges of all three biomarker values overlapped considerably between outcome groups and, therefore, could not independently distinguish between positive and negative findings.

**Figure 4:**
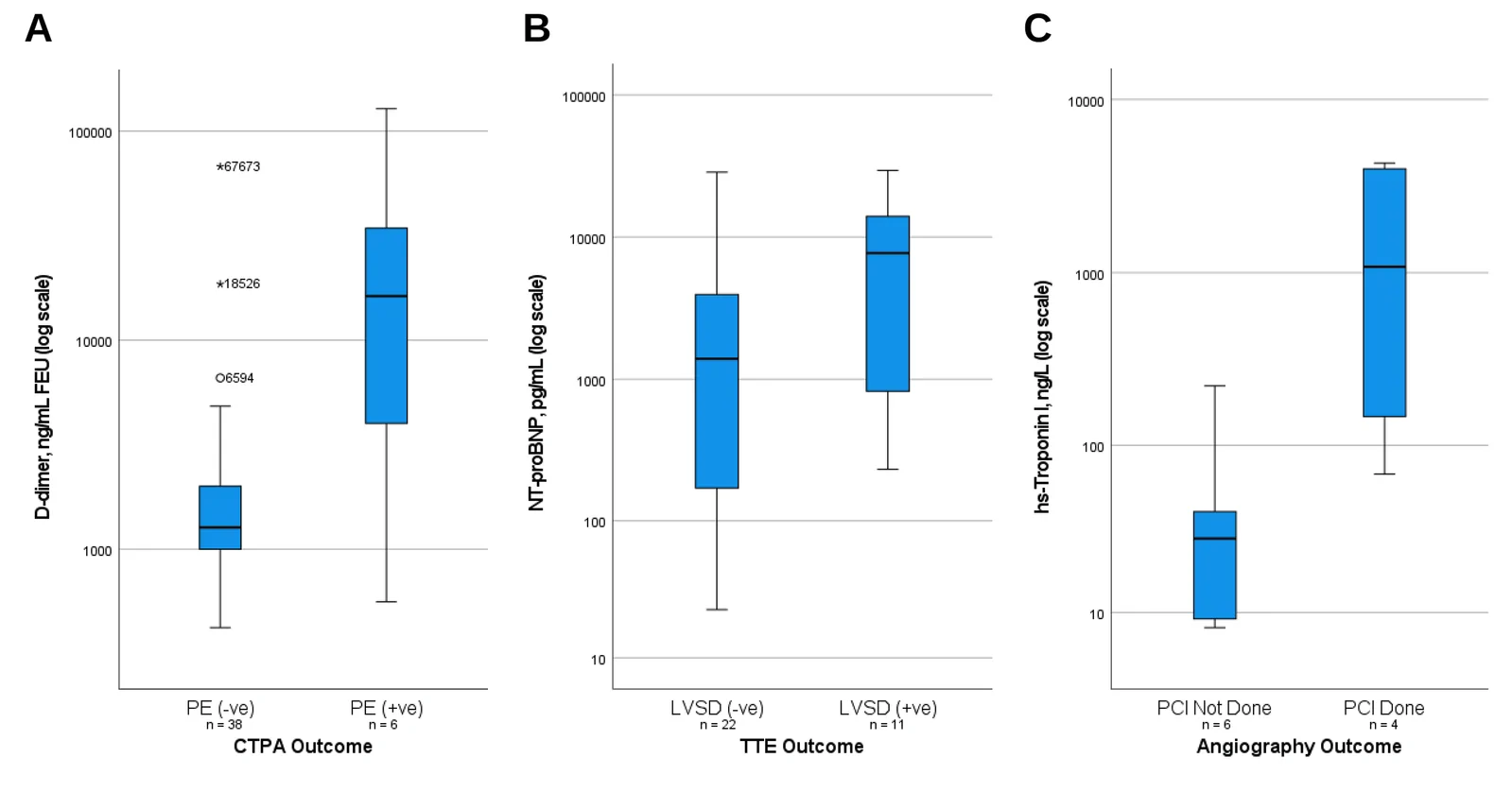
Biomarker concentrations by outcome in the PE (A), HF (B) and ACS pathways (C). Biomarker concentrations by outcome are displayed on a logarithmic scale: (A) D-dimer by PE status in patients undergoing CTPA for suspected PE (n = 44); (B) NT-proBNP by LVSD status in patients undergoing TTE (n = 33); and (C) hs-TnI by PCI status, as a proxy treatment outcome, in patients undergoing angiography for suspected ACS (n = 10). Boxes show the median and Tukey’s hinges; whiskers extend to 1.5 × the box length, calculated on the logarithmic scale. In panel C, the PCI median line appears at approximately 1,100 ng/L owing to its position on the logarithmic scale (arithmetic median, 1,997.4 ng/L). ACS: acute coronary syndrome; CTPA: computed tomography pulmonary angiography; HF: heart failure; hs-TnI: high-sensitivity troponin I; LVSD: left ventricular systolic dysfunction; NT-proBNP: N-terminal pro-B-type natriuretic peptide; PCI: percutaneous coronary intervention; PE: pulmonary embolism; TTE: transthoracic echocardiography. Image created in IBM SPSS Statistics for Windows, version 27.0 (released 2019; IBM Corp., Armonk, NY, USA).

Patient flow, biomarker testing, imaging, and outcomes across all three pathways are summarized in a single integrated schematic (Figure 5).

**Figure 5:**
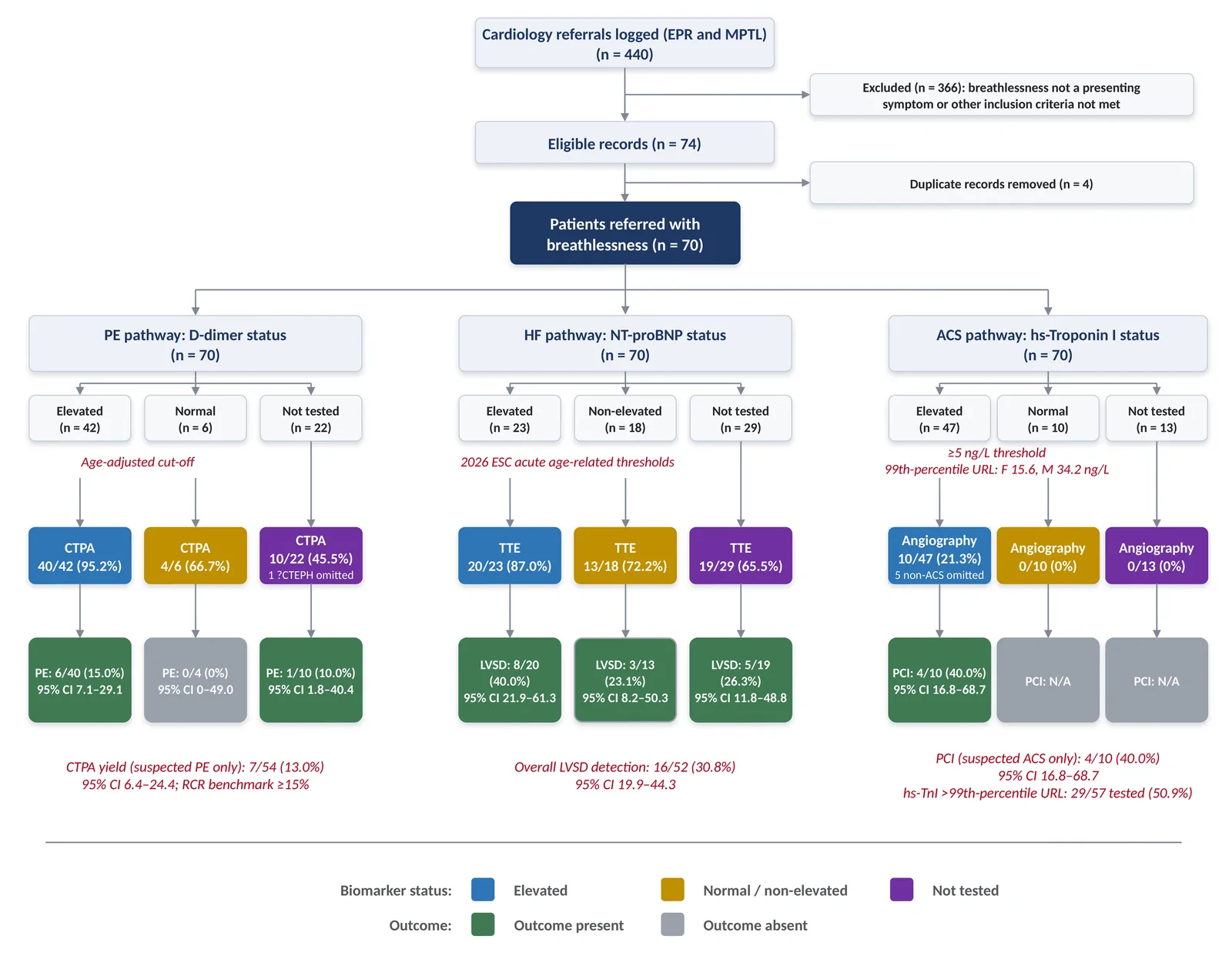
Integrated schematic of biomarker testing, imaging, and outcomes in the PE, HF, and ACS pathways. Overall patient flow from referral to biomarker status, imaging, and outcome, summarized in a single diagram. CTPA and coronary angiography boxes include only investigations performed for the relevant pathway indication (suspected PE and suspected ACS, respectively): one CTPA was performed for suspected chronic thromboembolic pulmonary hypertension (CTEPH), and five angiograms were performed for indications other than suspected ACS. All TTEs are included in the HF pathway, and all investigations are shown in Table 3. PCI is used as a proxy treatment outcome. ACS: acute coronary syndrome; CTPA: computed tomography pulmonary angiography; EPR: electronic patient record; HF: heart failure; hs-TnI: high-sensitivity troponin I; LVSD: left ventricular systolic dysfunction; MPTL: multiprofessional team list; NT-proBNP: N-terminal pro-B-type natriuretic peptide; PCI: percutaneous coronary intervention; PE: pulmonary embolism; TTE: transthoracic echocardiography; URL: upper reference limit Image created in Microsoft PowerPoint (Microsoft Corporation, Redmond, WA, USA).

## Discussion

Overall, the CTPA yield for suspected PE (13.0%) was numerically below the RCR standard of ≥15% [16] (Table 4). Almost all patients with an elevated D-dimer underwent CTPA (40/42, 95.2%), and the yield in this group (15.0%) matched the RCR benchmark, compared with 0% and 10.0% in patients with a normal or untested D-dimer, respectively. However, these differences were not statistically significant (p = 1.000), likely reflecting the limited statistical power afforded by the small comparator groups. This is consistent with D-dimer’s established role as a rule-out rather than an absolute discriminatory test, although the small sample size limits certainty. D-dimer was elevated in 87.5% of patients tested, reflecting its limited specificity in older, hospitalized patients, in whom age-related changes, infection, inflammation, and malignancy commonly raise levels independently of PE [5,7]. Notably, of the 14 patients with infection, our most common attributed cause of breathlessness (Table 2), 13 had a D-dimer measured, and all 13 results were elevated.

The pattern observed in our audit is not isolated: a South African single-center study reported a similar yield among patients with an elevated D-dimer (13.7%), also without a significant association between D-dimer status and PE [21], and a large UK tertiary center's own yield dipped marginally below the RCR threshold in one of four audited years [22]. This suggests that some fluctuation around the RCR benchmark may even be expected in well-established, large-scale pathways.

Wells score documentation fell well short of the RCR standard of 100% (12/54, 22.2%). By comparison, in the same UK audit, where Wells score entry was required on electronic CTPA requests, the score was missing in only 5% of requests, although a further 21% were discordant with their recorded criteria [22]. Such documentation gaps are addressable: mandating Wells score and D-dimer documentation at the point of CTPA request was associated with an absolute 6.6% increase in yield at a European center, with no PE among patients with both a low Wells score and a low D-dimer [23].

Among the 14 patients who did not undergo CTPA (excluding the incidental PE case), five (35.7%) reattended the hospital within 90 days, matching the follow-up window used in the Christopher Study and ADJUST-PE cohorts [4,5], for decompensated HF, pneumonia, atrial fibrillation, and nonrespiratory causes. Two died during the index admission from congestive HF, and one died after discharge within 90 days; the cause was unknown. No patient received a diagnosis of VTE. Two patients were started on therapeutic anticoagulation for new-onset atrial fibrillation. Although this would have treated any concurrent PE, it does not exclude an undiagnosed PE; hence, a missed diagnosis cannot be ruled out [7].

LVSD was detected more frequently with an elevated NT-proBNP than with a non-elevated result or no testing (40.0% vs 23.1% vs 26.3%), although neither difference was statistically significant (Table 5). The detection of LVSD in three patients with a non-elevated NT-proBNP is not unexpected, as the age-related thresholds applied are rule-in thresholds [19], whereas natriuretic peptides reliably exclude HF only below considerably lower rule-out cutoffs [11,24]. Moreover, most TTEs (32/52, 61.5%) were performed without an elevated biomarker result, which may reflect clinical suspicion of HF, although this audit could not assess this.

Although median NT-proBNP levels were higher in patients with LVSD than in those without LVSD (Figure 4B), NT-proBNP also rises with age, renal dysfunction, atrial fibrillation, infection, and anemia independently of left ventricular systolic function [10], several of which were common in our audit (Tables 1, 2). A raised NT-proBNP may therefore reflect a multitude of pathologies stemming from both cardiac and noncardiac etiologies [10].

Troponin was the most frequently tested biomarker and was elevated in 82.5% (47/57) of those tested, a proportion reflecting the use of the ≥5 ng/L threshold, as only 61.7% (29/47) of elevated results exceeded the sex-specific 99th-percentile URL. A high rate is unsurprising in an acutely breathless cohort, as dyspnea, cardiac disease, and systemic illness can overlap and raise troponin independently of ACS [25]. A previous retrospective study found troponin I positivity to be twice as frequent with breathlessness as with chest pain, with ACS accounting for only a few positive results [26]. Consistent with this, exceeding the URL did not, by itself, identify patients who underwent PCI: among the 10 patients undergoing angiography for suspected ACS, hs-TnI exceeded the URL in all four who underwent PCI and in four of the six who did not. In our audited population, angiography was performed in only a minority of patients with an elevated troponin (Table 3), indicating that it was undertaken selectively, consistent with the wide range of non-ACS causes of troponin elevation [12,26].

The substantial overlap in biomarker testing and imaging (Figures 1, 2) is consistent with the multifactorial nature of breathlessness, in which a significant proportion of patients have coexisting cardiac and noncardiac causes [27], although it may also reflect diagnostic uncertainty at presentation.

Building on our published conference abstract [18], recommendations arising directly from this audit relate to mandatory pretest documentation, targeted education, and reaudit. As none of the three RCR standards were met, and a Wells score was documented before CTPA in only 12/54 (22.2%) patients, this audit supports mandatory Wells score and D-dimer documentation at the point of a CTPA request, given the demonstrated association between mandatory documentation and improved diagnostic yield [23]. This audit also supports targeted education for ED, acute medical unit, and resident doctors, emphasizing, in line with national and European guidelines [6,7], that biomarker elevation alone does not establish a high pretest probability and should complement, rather than replace, a structured clinical assessment. A further recommendation based on existing guidance is the documentation of PERC in low-probability patients to avoid unnecessary D-dimer testing [8], although its age cutoff of <50 years limits its use in our cohort (median age 69.5 years). We propose a reaudit within 12 months of implementing these changes, against the RCR standards [16]: PE yield ≥15%, a clinically relevant alternative diagnosis in at least 50% of patients, and documented pretest probability in 100% of requests. A structured pathway with mandatory pretest documentation and targeted education may be a focus for future, larger multicenter studies, with the potential to improve diagnostic efficiency in identifying the etiology of an individual’s breathlessness.

Limitations

This audit only captured patients formally referred to cardiology with dyspnea as a primary or secondary symptom; those investigated for other reasons or through other routes were excluded. Hence, this sample is subject to selection bias, as cardiology-referred patients are likely to differ from the wider population undergoing CTPA in age, comorbidity burden, and pretest probability, and comparison of CTPA yield with the RCR standard should be regarded as indicative rather than direct. Patients with normal or untested biomarkers were imaged less often, so the reported yields are subject to partial verification bias and do not represent sensitivity, specificity, or predictive value. Biomarker results were linked to imaging within the same admission; all D-dimer and hs-TnI results preceded imaging, but a small number of NT-proBNP results were obtained after TTE. In the ACS pathway, PCI was used as a proxy treatment outcome that does not capture medically managed ACS, MINOCA, type 2 myocardial infarction, or referral for coronary artery bypass grafting. Furthermore, as all patients who underwent angiography for suspected ACS had an elevated hs-TnI, there was no comparator group. Several other comparator groups were small, so nonsignificant results may reflect a lack of statistical power rather than a true absence of difference, and all comparisons should be considered exploratory. Generalizability is further limited by the single-center setting and four-month audit period.

Further limitations arise from the retrospective data source, which depended on the completeness and accuracy of routine electronic documentation. A Wells score was documented before CTPA in only 12/54 patients, although scores may have been calculated without being recorded. The 90-day review of patients who did not undergo CTPA lacked a reference standard and was confined to records within our Trust, so missed diagnoses, including presentations to other hospitals unconnected to our Trust, cannot be excluded. Finally, systematic re-review identified four duplicate records and several data entry errors not detected during initial extraction; their correction revised the diagnostic yields relative to the conference abstract without changing the overall pattern of findings.

## Conclusions

Breathlessness does not always present with a clear cause; more often, several diagnoses can become plausible at once, reflecting its multifactorial nature and its potential to arise from dysfunction in multiple organ systems. This was mirrored by the substantial overlap in biomarker testing and imaging in this audit. Overall, the CTPA yield for suspected PE (13.0%, 95% CI 6.4-24.4) was numerically below the RCR benchmark of ≥15%. In the PE and HF pathways, biomarker elevation was accompanied by numerically higher yields, whereas no comparison was possible in the ACS pathway, as all patients undergoing angiography for suspected ACS had an elevated hs-TnI. The absence of statistical significance across these comparisons most plausibly reflects the small comparator groups available in this single-center audit. These findings should therefore be viewed as a descriptive, hypothesis-generating account of current practice rather than formal conclusions about biomarker performance or the effectiveness of pathways; confirmation in larger, multicenter studies with greater statistical power is needed to establish these findings. The identified documentation gaps highlight mandatory pretest documentation and targeted education as quality-improvement priorities, with reaudit within 12 months of their implementation against the RCR standards to evaluate their effect.
